# Late-onset and functional hypogonadism in aging men: an integrated perspective on hormonal, sleep, and metabolic dimensions

**DOI:** 10.1007/s40618-026-02981-6

**Published:** 2026-07-20

**Authors:** Monica Levy Andersen, J. M. Sanches, T. A. Alvarenga, M. Toricelli, M. B. Vasco, S. Tufik

**Affiliations:** 1 Departamento de Psicobiologia, Universidade Federal de São Paulo (UNIFESP) Rua Napoleão de Barros, 925 Vila Clementino, São Paulo, SP 04024-002 Brazil; 2 Instituto do Sono, Associação Fundo de Incentivo à Pesquisa, São Paulo, Brazil; 3 Centro de Ciências Naturais e Humanas, Universidade Federal do ABC (UFABC), São Paulo, Brazil; 4 Disciplina de Urologia, Universidade Federal de São Paulo (UNIFESP), São Paulo, Brazil

**Keywords:** Sleep, Testosterone, Andropause, Aging, Sleep disorders, Circadian rhythm, Metabolic syndrome, Hypogonadism, Obstructive sleep apnea, Testosterone replacement therapy

## Abstract

**Background and objective:**

Male reproductive aging is characterized by gradual hormonal and metabolic deterioration, often described as late-onset hypogonadism (LOH) or "andropause". However, these terms remain controversial and insufficient to capture the multifaceted nature of this process. This narrative review provides an integrated perspective on the interconnected hormonal, metabolic, sleep-related, and sexual consequences of male aging, with particular attention to the distinction between LOH and functional hypogonadism.

**Methods:**

We synthesized current literature on hormonal decline, sleep disturbances, and metabolic dysfunction in aging men, with a focus on physiological mechanisms, clinical manifestations, and integrative management strategies.

**Key findings and limitations:**

Progressive testosterone decline interacts with circadian disruption, sleep disorders, and metabolic imbalance in a bidirectional and clinically relevant manner. Unlike the abrupt hormonal changes in women, male decline is subtle, frequently underdiagnosed, and associated with reduced libido, fatigue, muscle loss, mood disturbances, and visceral adiposity. Sleep disturbances, including insomnia, sleep fragmentation, and obstructive sleep apnea, act both as consequences and amplifiers of endocrine and metabolic dysfunction. Current diagnostic criteria for LOH remain debated, limiting clinical recognition and delaying interventions. The distinction between LOH and functional hypogonadism is clinically meaningful, as the latter may be partially reversible through weight reduction, metabolic optimization, physical activity, and sleep-focused interventions. This review is narrative in nature, which may restrict systematic generalization of findings.

**Conclusions and clinical implications:**

An integrated perspective on male reproductive aging that incorporates hormonal, metabolic, and sleep-related dimensions supports earlier diagnosis and personalized therapeutic strategies, with implications for improving men’s health, quality of life, and longevity.

## Introduction

Late onset hypogonadism (LOH), also referred to as age-related testosterone deficiency, describes a clinical condition characterized by compatible symptoms in the presence of low circulating testosterone in older men. However, the diagnostic threshold for LOH is not universally accepted. Different societies and studies use distinct biochemical cutoffs, symptom requirements, and diagnostic algorithms, which contributes to substantial variability in prevalence estimates and clinical interpretation [1, 2]. The American Urological Association does not rely on a biochemical cutoff in isolation; its framework requires signs and symptoms consistent with hypogonadism together with low morning total testosterone, defined as a value below 300ng/dL confirmed on 2 separate occasions, measured under fasting conditions with a reliable assay [3]. The European Association of Urology follows a comparable symptom-based logic, but adopts a higher biochemical threshold of 12nmol/L (approximately 346ng/dL) as a potential criterion for initiating testosterone therapy in men presenting with hypogonadal symptoms, including reduced libido, erectile dysfunction (ED), and fewer morning erections [4]. Therefore, LOH should be interpreted as a heterogeneous and still debated construct rather than a uniformly defined entity [5, 6]. In this context, it is important to distinguish classical organic hypogonadism from functional hypogonadism. Functional hypogonadism refers to a potentially reversible suppression of the hypothalamic pituitary gonadal axis that occurs in the absence of structural abnormalities, commonly associated with obesity, insulin resistance, chronic inflammation, and sleep disorders. This concept is particularly relevant in aging men, in whom metabolic and sleep-related disturbances frequently coexist and may contribute to reductions in testosterone levels without primary gonadal failure. Thus, functional hypogonadism provides a useful framework to understand how metabolic dysfunction and sleep disruption interact with hormonal regulation, forming the basis for the integrated model explored in this review. The Massachusetts Male Aging Study estimated that 2.4 million American men between 40 and 69 years of age have androgen deficiency [2], while EMAS reported a 2.1% prevalence in European men between 40 and 79 years based on more restrictive criteria [5, 6]. Testosterone levels decline progressively with age, and up to 35% of men in their seventies show biochemical evidence of hypogonadism [1]. Only a subset of symptomatic men fulfill both biochemical and clinical criteria, and isolated symptoms without confirmed testosterone deficiency do not warrant a diagnosis [6, 7]. Sleep problems including insomnia, fragmented sleep, and obstructive sleep apnea (OSA) are frequently observed in aging men, but remain underrecognized within the clinical context of LOH. These issues compromise quality of life and may worsen the hormonal and metabolic imbalances that accompany testosterone decline. The mechanisms underlying LOH are multifactorial. Primary hypogonadism results from testicular failure and is characterized by low testosterone and elevated gonadotropin levels. Secondary hypogonadism originates from hypothalamic or pituitary dysfunction and is often linked to excess body weight and metabolic abnormalities [8, 9]. EMAS data indicated that 73% of men with LOH were overweight or obese, with only a minority presenting with idiopathic causes [9]. A third form, known as compensated hypogonadism, is defined by normal testosterone alongside elevated luteinizing hormone (LH) and may represent a transitional or adaptive state [10]. Testosterone replacement therapy (TRT) remains the standard treatment for confirmed cases and is available through several delivery methods [11]. However, inappropriate use is widespread. Up to one-third of men receiving TRT do not meet the biochemical criteria for hypogonadism, and many lack proper clinical follow-up [12, 13]. In this context, causal pathways are difficult to disentangle, as obesity, insulin resistance, inflammation, and testosterone deficiency may act as both drivers and consequences within the same pathophysiological network. Clarifying how testosterone deficiency contributes to metabolic dysfunction is critical for guiding future interventions aimed at preserving health during male aging. This review examines the metabolic consequences of LOH, with particular attention to the role of sleep disturbances. LOH refers to a debated clinical endocrine diagnosis, whereas functional hypogonadism denotes a potentially reversible mechanistic subtype driven by obesity, insulin resistance, chronic inflammation, and sleep disorders. Both constructs are addressed throughout this review within an integrative perspective on the multisystem consequences of male aging.

## Methods

This study was designed as a narrative review. A PubMed search was conducted for studies published between 1998 and 2025 using combinations of MeSH terms and keywords related to andropause, male aging, testosterone deficiency, late-onset hypogonadism, functional hypogonadism, metabolism, sleep, obstructive sleep apnea, erectile dysfunction, and inflammation. A representative core search strategy was: “andropause” AND “male” AND “aging” AND “metabolism”. Studies were selected according to their relevance to the endocrine, metabolic, sleep-related, and sexual dimensions of male aging. Articles not available in English, studies without full-text access, and case reports were excluded. Given the narrative nature of this review, the objective was not to perform a systematic appraisal of all available evidence, but to provide a structured and clinically oriented synthesis of the literature most relevant to the integrative perspective discussed in this manuscript.

### Clinical outcomes of testosterone decline in aging males

Testosterone production declines with aging. The European Male Aging Study (EMAS) reported a 0.4% per annum (log hormone-age) decrease in total testosterone and a 1.3% per annum decline in free testosterone [7]. Within this perspective, 2 clinically relevant constructs are considered in parallel: LOH, a debated endocrine diagnosis characterized by clinical symptoms and metabolic alterations that significantly impact men’s health and quality of life, and functional hypogonadism, a potentially reversible mechanistic subtype linked to obesity, insulin resistance, chronic inflammation, and sleep disorders. This framing supports the discussion of clinical outcomes presented in the following sections.

### Age-related decline in testosterone and its clinical implications

Testosterone levels decline progressively with age, typically beginning in the mid-30s. This reduction, particularly in free testosterone due to increased sex hormone-binding globulin (SHBG), underlies a constellation of symptoms collectively referred to as LOH [14]. Clinical manifestations include reduced libido, ED, fatigue, decreased muscle mass and strength, visceral fat accumulation, low bone density, and mood and cognitive disturbances [15–17]. These symptoms vary in intensity among individuals and are influenced by comorbidities, such as obesity, metabolic syndrome, and chronic illnesses, which may amplify the impact of androgen deficiency [18, 19].

Sleep disturbances are commonly reported in men with LOH and may further suppress testosterone secretion. Testosterone follows a circadian rhythm and peaks during slow-wave sleep [20]. Partial sleep deprivation alone has been shown to lower testosterone levels within a few days, suggesting a bidirectional relationship between sleep and endocrine health [21]. Low testosterone levels have been correlated with increased cardiovascular risk, including endothelial dysfunction and arterial stiffness, which are considered early markers of atherosclerosis [22, 23]. Meta-analyses support an association between low endogenous testosterone and increased all cause and cardiovascular mortality in men. Observational studies suggested that restoration of testosterone to physiological levels may be related to lower mortality and fewer major cardiovascular events. Causal interpretation remains cautious, since much of the evidence was observational, definitions of hypogonadism vary across studies, and randomized trials to date have more consistently demonstrated cardiovascular safety or neutrality of testosterone therapy than a definitive reduction in mortality [23–26].

In men with obesity and type 2 diabetes (T2D), reduced testosterone levels often reflect functional hypogonadism, a potentially reversible suppression of the hypothalamic pituitary testicular axis, rather than irreversible organic hypogonadotropic hypogonadism. In this setting, visceral adiposity, insulin resistance, chronic low grade inflammation, and altered adipokine signaling interact to impair gonadotropin secretion and testosterone production [26]. This distinction is clinically important because low testosterone in these men may represent both a marker and a contributor to metabolic dysfunction, reinforcing a bidirectional relationship between endocrine and cardiometabolic health [26]. Men with prediabetes and accompanying metabolic disturbances may present with symptoms commonly attributed to androgen deficiency, including reduced energy, depressive complaints, and sexual symptoms, particularly during midlife [27]. These findings highlight the importance of a comprehensive endocrine and metabolic evaluation in men with metabolic risk factors, as identifying functional hypogonadism may open opportunities for targeted interventions focused on weight reduction, metabolic control, and management of connected comorbidities, alongside careful consideration of hormonal therapy when appropriate.

Prolactin should be considered in the evaluation of male hypogonadism, particularly when secondary hypogonadism is suspected. Hyperprolactinemia may suppress hypothalamic gonadotropin-releasing hormone (GnRH) secretion, leading to reduced LH release and impaired testosterone production [28]. In clinical practice, elevated prolactin may contribute not only to biochemical hypogonadism, but to low sexual desire, erectile complaints, and other manifestations of sexual dysfunction [28]. Recognizing this mechanism is important because it expands the differential diagnosis of testosterone deficiency beyond aging and metabolic dysfunction alone.

Sexual dysfunction, especially ED, becomes more prevalent with age and is often linked to declining testosterone levels [29]. Testosterone replacement therapy tends to improve libido and overall sexual activity more consistently than erectile function itself, likely because erection depends on a broader set of mechanisms that extend beyond androgen status, including vascular integrity, endothelial nitric oxide signaling, intact neural pathways, psychological factors, and age-related comorbidities, such as obesity, type 2 diabetes, cardiovascular disease, and sleep disorders [30]. Hypogonadism may contribute to PDE5 inhibitor treatment failure in some men, particularly when reduced testosterone is accompanied by diminished sexual desire, fewer nocturnal erections, and impaired androgen support of penile tissue structure and nitric oxide dependent pathways [28]. These findings reinforce the multifactorial etiology of ED and support individualized therapeutic approaches that combine hormonal evaluation with management of comorbidities and optimization of ongoing oral therapy. Taken together, the clinical consequences of age-related testosterone decline are complex and modulated by metabolic, inflammatory, and lifestyle factors. Understanding this interplay is essential for tailoring interventions. These may include TRT when appropriate, alongside the management of comorbidities and targeted lifestyle modifications.

### Testosterone, metabolic homeostasis, and tissue crosstalk in aging men

Testosterone plays a central role in maintaining metabolic homeostasis by acting on multiple organs, including skeletal muscle, adipose tissue, the liver, and pancreas. In skeletal muscle, testosterone enhances glucose uptake by promoting GLUT4 translocation and increases insulin sensitivity, thereby improving glycemic control [20]. In adipose tissue, it inhibits lipid accumulation by downregulating lipoprotein lipase activity and stimulating lipolysis, reducing visceral fat, a key feature of metabolic syndrome [20, 31]. In the liver, testosterone modulates gluconeogenesis and lipid metabolism by regulating the expression of enzymes, involving phosphoenolpyruvate carboxykinase and hormone-sensitive lipase [31]. These coordinated actions contribute to the maintenance of energy balance and reduce the risk of insulin resistance and dyslipidemia. Testosterone influences pancreatic β-cell function. Human β-cells express steroidogenic enzymes, among them 5α-reductase and aromatase, enabling the local conversion of testosterone into dihydrotestosterone (DHT) and estradiol. This intracrine metabolism is essential for potentiating glucose-stimulated insulin secretion (GSIS) and for protecting β-cells against oxidative stress [32, 33]. The study by Xu et al. (2020) established that blocking either DHT or estradiol synthesis impairs the insulinotropic effect of testosterone, highlighting the relevance of local steroid metabolism within β-cells [32]. In experimental models, testosterone deficiency impairs insulin signaling pathways in skeletal muscle, whereas supplementation restores insulin sensitivity through activation of PI3K and AKT [34, 35]. Longitudinal studies showed that lower baseline levels of total and bioavailable testosterone predict higher HOMA-IR scores over time, independent of confounders [36]. Reverse causality appears less likely, as higher insulin resistance at baseline did not predict testosterone decline in follow-up analyses [36].

In lipid metabolism, testosterone and its receptor (AR) influence mitochondrial function, lipid oxidation, and the expression of lipogenic genes. Testosterone deficiency is associated with dyslipidemia, characterized by elevated triglycerides and LDL cholesterol, reduced HDL levels, and increased visceral adiposity [37, 38]. Recent studies underscore the role of androgens in regulating mitochondrial morphology and function, which impacts energy metabolism and lipid oxidation [39, 40]. Polymorphisms, like APOE isoforms, modulate the lipid-related effects of testosterone deficiency, exacerbating proatherogenic profiles in certain genotypes [39, 40]. *In vitro* data confirm that testosterone suppresses the expression of lipogenic genes, comprising FASN and SREBP-1 in hepatocytes, further supporting its protective role against lipid accumulation and vascular damage [41, 42]. Testosterone regulates protein metabolism in skeletal muscle by promoting protein synthesis and reducing degradation. This effect becomes particularly relevant during energy deficits or aging-related sarcopenia [43]. In states of hypogonadism, the interaction between testosterone and insulin is disrupted, impairing anabolic processes and contributing to muscle wasting [44]. Supplementation may restore anabolic capacity and enhance insulin signaling in muscle cells, highlighting its potential role in preserving functional capacity in older men [35]. These findings position testosterone as a systemic metabolic regulator whose decline during aging contributes to a broad range of metabolic dysfunctions, including impaired insulin action, dyslipidemia, and unfavorable body composition. Understanding these mechanisms is essential for developing targeted strategies to monitor, prevent, or mitigate metabolic complications in aging men.

### Interplay between testosterone decline and sleep in aging men

Testosterone, the primary androgenic hormone in men, follows a distinct circadian rhythm and is closely linked to sleep quality and its different stages. Research indicated that testosterone secretion peaks during the deeper phases of sleep, particularly slow wave sleep (SWS), and to a lesser extent during rapid eye movement (REM) sleep [45, 46]. Disruptions in sleep patterns, such as insomnia, sleep fragmentation, or sleep restriction, can significantly impair this process, leading to reduced circulating testosterone levels [47, 48]. This close physiological coupling helps explain why even mild sleep disturbances can disrupt hormonal function in young, otherwise healthy individuals.

The interplay between testosterone decline and sleep architecture in aging men is complex. Testosterone influences sleep architecture by modulating central nervous system mechanisms involved in circadian and homeostatic regulation. With aging, its decline has been related with changes in sleep macrostructure, including decreased SWS and REM sleep, increased sleep latency, and frequent nighttime awakenings [49–51]. Experimental sleep restriction studies in healthy men showed a 10 to 15% reduction in serum testosterone levels after just one week of partial sleep deprivation, despite stable LH levels, suggesting a direct suppressive effect of poor sleep on androgen production [21, 52].

Multiple lines of evidence converge on a clinically relevant interaction between OSA, testosterone status, and erectile function in aging men. Evidence from cross-sectional and longitudinal studies points to higher rates of ED and lower serum testosterone in men with moderate-to-severe OSA compared with men without sleep-disordered breathing, with the magnitude of the relationship scaling with apnea severity and concurrent metabolic burden [53, 54]. Within this body of evidence, cross-sectional analyses of 2 editions of the EPISONO sleep study (2007 and 2018) in São Paulo identified a significant relashionship between OSA (AH≥15 events/hour) and ED, with advancing age and lower testosterone levels independently correlated with increased ED risk [55, 56]. These findings are consistent with international epidemiological data connecting OSA severity to androgen status and erectile function in aging men [57]. The available evidence supports a multifactorial and potentially bidirectional connection between sleep disturbances, hormonal decline, and sexual dysfunction, in which OSA may both contribute to reduced testosterone and ED and serve as a clinical marker of broader age-related physiological changes. Causal inference nonetheless remains limited by the frequent coexistence of sleep disruption, obesity, metabolic syndrome, and hypogonadal symptoms across study populations, overlapping facets of the multisystem deterioration of the aging man, and by methodological heterogeneity in the definition of hypogonadism and ED assessment between studies.

The age-related decline in testosterone is closely tied to disruptions in the hypothalamic-pituitary-gonadal (HPG) axis, which is regulated by circadian pacemakers in the suprachiasmatic nucleus [58]. With advancing age, this rhythm becomes blunted, leading to a lower frequency and amplitude of hormone release, which contributes to symptoms implicated to hormonal decline. These alterations are commonly seen in individuals with metabolic syndrome and may intensify in aging men with declining androgen levels [59, 60]. The disruption of circadian organization contributes to the deterioration of metabolic function, reinforcing the endocrine imbalance that characterizes male aging.

Sleep disturbances are prevalent in older men and represent another important factor influencing this stage of life. OSA affects a large portion of this population and shows a consistent link with reduced testosterone levels [56, 61]. Evidence indicated that lower androgen levels compromise the tone of upper airway muscles and ventilatory stability, which may increase the risk for OSA. In addition to OSA, insomnia, restless legs, and excessive sleepiness during the day are frequently reported by aging men and often coexist with emotional changes and metabolic impairments. Mood disturbances may further intensify this cycle, since depression and anxiety can impair sleep, whereas chronic sleep disruption may worsen emotional symptoms. Low testosterone may contribute to fatigue, low mood, and reduced motivation, reinforcing the overlap between endocrine, sleep, and psychological complaints in aging men [62, 63]. These symptoms resemble those experienced by women after menopause, when the decline in estrogen and progesterone leads to similar disruptions in sleep regulation and energy balance. Both testosterone and estrogen influence neural circuits involving GABA, serotonin, and dopamine, and contribute to the regulation of stress and inflammatory responses [64]. Interventional data on whether treatment of OSA improves gonadal function remain less consistent than the observational evidence connecting OSA to lower testosterone levels [65]. Meta-analyses of CPAP studies have generally reported neutral effects on circulating testosterone and gonadotropins, suggesting that correction of sleep disordered breathing does not necessarily restore hormonal status in all men [65, 66]. These observations are important because they caution against overstating a direct causal pathway between OSA and hypogonadism. At the same time, emerging evidence from selected populations, including men with severe obesity, suggests that CPAP induced hormonal improvement may occur in some settings, although these data remain limited and heterogeneous [67].

Reduced sleep quality and circadian misalignment impair melatonin secretion. This hormone plays an essential role in maintaining energy metabolism, protecting mitochondria from oxidative damage, and regulating glucose handling [68]. Declining melatonin levels may therefore contribute to oxidative stress, inflammation, and insulin resistance in aging men, compounding the effects of androgen deficiency. Sleep health should be regarded as a central component in the evaluation and care of aging men, not only for its direct physiological effects, but for its interplay with endocrine and metabolic systems. Understanding these mechanisms is key to developing effective strategies to mitigate the multisystem impact of male aging on health and quality of life [69].

### Inflammation and hormonal decline

In a cross-sectional study of healthy nonsmoking men, aging, especially when accompanied by lower testosterone levels, coincided with impaired endothelial function, higher inflammatory markers, and greater oxidative stress [70]. Among older participants, those with lower testosterone had worse flow mediated dilation than those with higher testosterone, and antioxidant infusion improved vascular function only in the low testosterone group, supporting a role for oxidative stress in this dysfunction [70]. Inflammatory activity was likewise more pronounced in men with lower testosterone, with higher IL-6 and CRP levels and an inverse relationship between these markers and endothelial performance [70]. Sleep deprivation may further worsen this profile by increasing proinflammatory cytokines, including IL-6 and TNF-α, which are known to inhibit testosterone synthesis and may intensify the inflammatory and endocrine imbalance observed in aging men [70]. This evidence highlight a complex interaction between hormonal, vascular, and inflammatory systems that characterize the male aging process. In this context, declining testosterone is not only a marker of aging, but a contributor to systemic dysfunction.

Bone health is significantly affected by hormonal and inflammatory changes during male aging. Testosterone supports bone mineralization both directly, through androgen receptor signaling in osteoblasts, and indirectly, through aromatization to estradiol, which is the principal regulator of bone resorption in men. Hypogonadism, whether organic or functional, is a recognized contributor to reduced bone mineral density, osteoporosis, and fragility fractures in older men, and the magnitude of this risk has been characterized both in primary studies and in dedicated reviews on male skeletal aging [71–73].

Testosterone plays an immunomodulatory role by inhibiting pro-inflammatory cytokines, including TNF-α, IL-1β, and IL-6, while enhancing IL-10. A randomized, placebo-controlled crossover trial in hypogonadal men showed that TRT reduced TNF-α and IL-1β and increased IL-10, shifting the balance toward an anti-inflammatory profile [74]. Chronic inflammation, in turn, suppresses testosterone production by disrupting the HPG axis and impairing Leydig cell function. *In vitro* studies with TM3 Leydig cells demonstrated that exposure to IL-1β, TNF-α, and IL-6 suppressed testosterone synthesis in a dose-dependent manner [75]. TRT has been shown to mitigate inflammation and improve metabolic health. A 12-month intervention in hypogonadal men led to reductions in BMI, fat mass, leptin, and CRP, alongside increased adiponectin [76]. Similarly, TRT in hypogonadal men with metabolic syndrome resulted in lower levels of insulin, IL-1β, TNF-α, and CRP after 30 weeks [77]. These studies suggested that restoring testosterone may break the feedback loop between hypogonadism and systemic inflammation, improving both endocrine and metabolic outcomes. The data support a bidirectional relationship between testosterone deficiency and inflammation. Hormonal decline fuels systemic inflammation, which in turn suppresses testosterone production. Disrupting this cycle through TRT and lifestyle interventions offers a promising strategy to improve vascular, metabolic, and bone health in aging men.

### Toward an integrated understanding of testosterone decline in aging men

Although the reduction in testosterone with aging is well established, its clinical relevance varies significantly across individuals. Importantly, the presence of symptoms typically linked to androgen deficiency, including sexual dysfunction, reduced muscle mass, depressive mood, fatigue, and metabolic changes, does not consistently align with serum testosterone levels. This disconnect challenges the adequacy of a purely biochemical definition of hypogonadism in older men and supports a shift toward a symptom-centered and integrative diagnostic framework.

A major difficulty in managing testosterone deficiency lies in distinguishing between normal aging processes and the pathological features of hypogonadism. Many of the symptoms attributed to low testosterone overlap with those of chronic conditions including T2D, cardiovascular disease, and mood disorders. This overlap raises a fundamental question: is testosterone deficiency a cause, a consequence, or simply a biomarker of systemic decline? The answer remains elusive, especially in light of the bidirectional relationship between testosterone levels and metabolic health. This interdependence complicates treatment decisions, particularly in men with multiple comorbidities where TRT may offer symptomatic relief yet carries potential risks. To navigate this complexity, current clinical guidelines emphasize diagnostic rigor and individualized care. The Endocrine Society recommends testosterone replacement only for men who present with consistent symptoms and confirmed low testosterone levels on repeated early-morning assessments. The American Urological Association stresses the importance of individualized risk-benefit analysis, particularly in men with cardiovascular disease or a history of prostate cancer. The European Association of Urology and the International Society for Sexual Medicine have supported these principles while advocating for broader evaluations of health-related quality of life, beyond sexual function alone.

The therapeutic landscape has expanded beyond injectable esters to include transdermal gels, patches, intranasal formulations, and long-acting subcutaneous options. These modalities seek to improve hormonal stability and patient adherence. However, long-term safety data remain limited, especially in high-risk cardiovascular populations. Although recent randomized trials, including the TRAVERSE study [22], have yielded promising safety outcomes in selected groups, earlier observational studies reported conflicting results, reinforcing the need for cautious, case-by-case evaluation.

A growing body of evidence supports the existence of functional hypogonadism, particularly in the setting of obesity, metabolic syndrome, and chronic low-grade inflammation. In these cases, the hypothalamic-pituitary-testicular axis may be reversibly suppressed. Interventions, such as weight loss, regular physical activity, glycemic control, and sleep optimization have shown potential to restore endogenous testosterone production without pharmacological replacement. This distinction between testosterone’s role as a physiological hormone and its use as a medical therapy is essential. Exogenous administration fails to reproduce the circadian pulsatility of natural testosterone secretion and may suppress spermatogenesis, increase hematocrit, alter lipid profiles, and impair long-term reproductive and metabolic health.

**Fig. 1:**
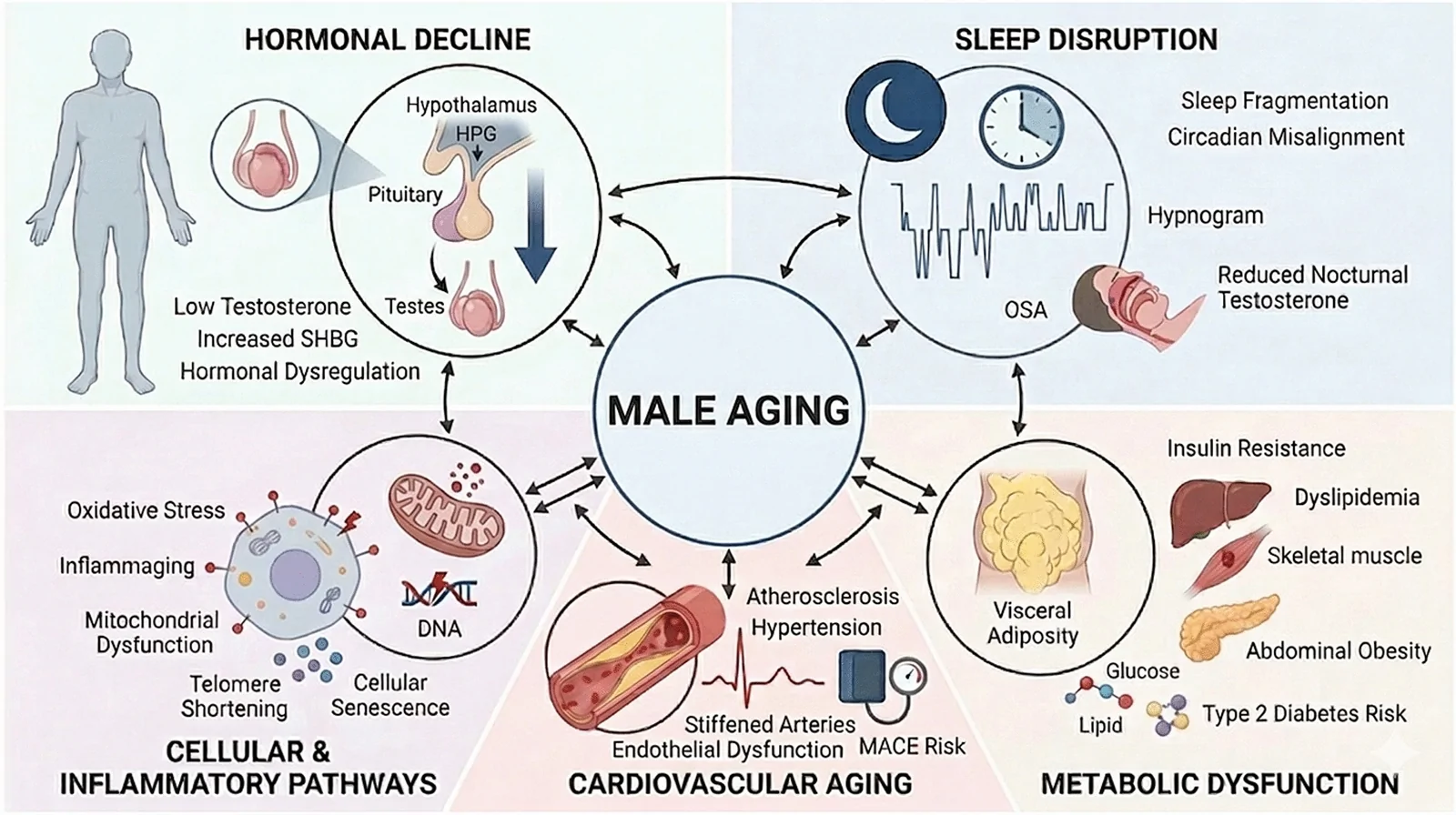
Integrated conceptual framework of multisystem male aging. This schematic illustration summarizes the multidirectional interactions among the main domains involved in male aging. Age-related hormonal decline, characterized by low testosterone, increased sex hormone-binding globulin, and dysregulation of the hypothalamic-pituitary-gonadal axis, interacts bidirectionally with sleep disruption, including sleep fragmentation, circadian misalignment, obstructive sleep apnea, and reduced nocturnal testosterone secretion. These alterations are closely linked to metabolic dysfunction, particularly visceral adiposity, insulin resistance, dyslipidemia, abdominal obesity, and increased risk of type 2 diabetes. In parallel, cellular and inflammatory pathways, including oxidative stress, inflammaging, mitochondrial dysfunction, telomere shortening, DNA damage, and cellular senescence, contribute to vascular injury and cardiovascular aging. These interconnected mechanisms promote endothelial dysfunction, arterial stiffness, hypertension, atherosclerosis, and increased risk of major adverse cardiovascular events, reinforcing the multisystem nature of male aging. Abbreviations: SHBG, sex hormone-binding globulin; HPG, hypothalamic-pituitary-gonadal; OSA, obstructive sleep apnea; DNA, deoxyribonucleic acid; MACE, major adverse cardiovascular events

Mechanistic insights reinforce testosterone’s central role in energy metabolism and systemic regulation. In skeletal muscle, it stimulates protein synthesis, enhances glucose uptake, and facilitates fatty acid oxidation through androgen receptor signaling. These effects promote muscle anabolism and improve insulin sensitivity. In adipose tissue, testosterone reduces fat cell formation and visceral fat accumulation by promoting lipolysis and limiting adipocyte differentiation. In the liver, it inhibits lipogenesis and supports insulin signaling, helping prevent non-alcoholic fatty liver disease. In pancreatic beta cells, which express androgen receptors, testosterone improves insulin secretion and protects against oxidative and lipotoxic damage. It supports vascular function by enhancing endothelial nitric oxide availability and reducing inflammatory signaling. In bone, testosterone promotes mineralization by stimulating osteoblast activity and, through aromatization to estradiol, contributes to the maintenance of bone density. Its age-related decline correlates with increased risk of osteoporosis and fragility fractures. These interconnected actions illustrate that testosterone is not solely a sex hormone, while is a critical regulator of multisystem homeostasis. The role of sleep cannot be overlooked. Testosterone secretion follows a diurnal pattern that is tightly linked to sleep architecture, particularly slow-wave sleep and REM sleep stages. Aging men often experience disrupted sleep, reduced sleep efficiency, and higher prevalence of sleep disorders, including insomnia and OSA. These changes not only impair quality of life, instead contribute to hormonal dysregulation. Poor sleep has been associated with decreased morning testosterone levels, reduced insulin sensitivity, elevated inflammatory cytokines, and impaired cognitive and emotional processing. Excessive sleepiness, low energy, and mood disturbances may be misinterpreted as isolated symptoms of hypogonadism when they in fact originate from sleep fragmentation or circadian misalignment. Integrating sleep evaluation into the assessment of aging men may help identify reversible contributors to symptoms attributed to testosterone deficiency. In some cases, treating sleep disorders may enhance response to testosterone therapy or reduce the perceived need for pharmacological intervention. This reinforces the necessity of a comprehensive clinical model that accounts for neuroendocrine, metabolic, vascular, skeletal, and behavioral dimensions of male aging (Fig. 1).

Ultimately, testosterone decline in aging men is best understood not only as an endocrine phenomenon, but as part of a broader physiological transition. Future management of male aging should be individualized and multidisciplinary, integrating lifestyle intervention, treatment of reversible contributors, and, when appropriate, carefully selected hormonal therapy. This distinction is especially relevant in functional hypogonadism, in which weight reduction, physical activity, metabolic optimization, and sleep improvement may help restore endogenous testosterone production, whereas men with unequivocal organic hypogonadism may require more direct hormonal treatment. Available therapeutic options span different formulations and regimens, allowing management to be tailored to clinical profile, comorbidities, reproductive goals, and patient preference. This integrative approach requires clinicians to consider sleep health, metabolic resilience, cardiovascular integrity, musculoskeletal maintenance, and psychological well-being alongside hormonal assessment (Table 1). Several unresolved issues remain in this field, including the heterogeneity of diagnostic definitions for testosterone deficiency, the difficulty in disentangling causality from findings observed in men with obesity, sleep disorders, and metabolic syndrome, and the limited comparability between observational and interventional studies. Future research should prioritize standardized phenotyping, longer controlled studies, and integrated models capable of capturing the bidirectional interactions among endocrine, metabolic, sleep-related, and sexual domains. Clinically, aging men should be made aware that hormonal, metabolic, sleep-related, and sexual changes rarely occur in isolation, and that their early recognition is essential to prevent progression into the multisystem impairment of the aging man.

**Table 1:** Integrated domains involved in male aging and their clinical relevance

Domain	Main alterations	Potential mechanisms	Clinical manifestations	Clinical implications
Hormonal decline	Progressive reduction in total and especially free testosterone with aging	Increased SHBG, reduced gonadal output, hypothalamic pituitary testicular axis dysregulation	Reduced libido, fatigue, reduced muscle mass, low bone density, mood changes	Requires careful distinction between physiological aging, LOH, and functional hypogonadism
Sleep disturbance	Insomnia, sleep fragmentation, reduced slow wave sleep, OSA, circadian disruption	Impaired nocturnal testosterone secretion, HPG axis dysregulation, altered melatonin rhythms	Daytime sleepiness, low energy, cognitive complaints, worsening sexual symptoms	Sleep assessment should be part of the evaluation of aging men with suspected testosterone deficiency
Metabolic dysfunction	Visceral adiposity, insulin resistance, dyslipidemia, altered glucose homeostasis	Reduced androgen signaling in muscle, adipose tissue, liver, and pancreas; chronic low grade inflammation	Weight gain, metabolic syndrome, T2D, worsening cardiometabolic profile	Supports recognition of bidirectional links between low testosterone and metabolic impairment
Inflammation and oxidative stress	Higher IL 6, TNF α, CRP, endothelial dysfunction	Chronic inflammation suppresses testosterone production and worsens vascular function	Fatigue, vascular impairment, metabolic deterioration	Reinforces the need for integrated endocrine and cardiometabolic assessment
Sexual health impairment	Reduced sexual desire, ED, poorer response to PDE5 inhibitors in some men	Low androgen support, endothelial dysfunction, neural and vascular contributors, comorbidities	Lower libido, fewer nocturnal erections, erectile complaints	Sexual symptoms should be interpreted within a broader endocrine, vascular, and sleep-related context
Functional hypogonadism	Low testosterone in the setting of obesity, T2D, metabolic syndrome, and sleep disorders	Potentially reversible suppression of the HPG axis without structural gonadal disease	Symptoms overlapping with androgen deficiency and metabolic disease	May improve with weight reduction, metabolic optimization, physical activity, and sleep focused interventions
Therapeutic perspective	Lifestyle measures, treatment of reversible contributors, selected hormonal therapy when indicated	Multidisciplinary management according to clinical profile and etiology	Variable symptomatic improvement depending on phenotype and comorbidities	Management should be individualized, not limited to a replacement versus non replacement model

CRP, C-reactive protein; ED, erectile dysfunction; HPG, hypothalamic-pituitary-gonadal; IL-6, interleukin-6; LOH, late-onset hypogonadism; OSA, obstructive sleep apnea; PDE5, phosphodiesterase type-5; SHBG, sex hormone binding globulin; T2D, type 2 diabetes; TNF-α, tumor necrosis factor alpha
